# Sex-based differences in outcomes following endovascular therapy for anterior circulation large vessel occlusion: a pooled analysis of DEVT, RESCUE BT, and MARVEL trials

**DOI:** 10.3389/fneur.2025.1753257

**Published:** 2026-01-12

**Authors:** Yangyang Duan, Jinfu Ma, Xu Xu, Haoxuan Zhu, Xiaolei Shi, Shihai Yang, Zhixi Wang, Mingyang Chen, Yihui Yang, Yuhan Fan, Binghan Wang, Guojian Liu, Linyu Li, Zhenxuan Tian, Boyu Chen, Chawen Ding, Dahong Yang, Wenzhe Sun, Gaoming Li, Lilan Wang, Shitao Fan, Chengsong Yue, Nizhen Yu, Jie Yang, Wei Li, Zhuang Li, Lingyu Zhang

**Affiliations:** 1 Department of Neurology, The First Affiliated Hospital of Hainan Medical University, Haikou, China; 2 Department of Neurology, Xinqiao Hospital and The Second Affiliated Hospital, Army Medical University (Third Military Medical University), Chongqing, China; 3 Department of Neurology, The 908th Hospital of Chinese People’s Liberation Army Joint Logistic Support Force, Nanchang, China; 4 Department of Neurology, The Second Affiliated Hospital of Chongqing Medical University, Chongqing, China

**Keywords:** acute ischemic stroke, clinical outcome, endovascular treatment, large vessel occlusion, sex

## Abstract

**Background:**

This study sought to characterize sex-specific treatment effects by comparing clinical outcomes between men and women undergoing EVT.

**Methods:**

Analyses were based on the DEVT, RESCUE BT, and MARVEL databases. Men and women were matched using propensity score matching (PSM). The primary outcome was defined as the 90-day ordinal modified Rankin Scale score (mRS) distribution. Secondary outcomes included the favorite outcome (mRS 0 to 3), functional independence (mRS 0 to 2), and excellent outcome (mRS 0 to 1). Safety outcomes were symptomatic intracranial hemorrhage (sICH) and mortality.

**Results:**

Of 2,862 patients, 1,221 (42.7%) were women and 1,641 (57.3%) were men. After adjusting for covariates, there were no sex differences in 90-day ordinal mRS distribution (median [interquartile range], 3 [1–6] versus 3 [1–5], common odds ratio [OR], 1.02 [0.89–1.18], *p* = 0.741). The secondary outcomes demonstrated consistency with the primary findings, and the safety outcomes remained stable across men and women. After 1:1 PSM, the results remained consistent with the adjusted outcomes described above.

**Conclusion:**

This pooled analysis demonstrated that no statistically significant differences were observed between men and women in clinical or safety outcomes following EVT for anterior circulation LVO. Furthermore, there was no evidence of interaction between sex and predefined subgroups in terms of treatment effect modification for EVT outcomes.

## Introduction

Ischemic stroke caused by large vessel occlusion (LVO) in the anterior circulation is a leading cause of disability and mortality worldwide (1). Over the past decade, endovascular treatment (EVT) has become the optimal therapy for eligible patients, as multiple landmark randomized controlled trials have demonstrated superior recanalization rates and functional independence compared to medical therapy alone (2–5). Despite significant technical advancements in EVT, clinical outcomes continue to demonstrate substantial heterogeneity across the patient population. This variability has motivated extensive research into potential determinants of treatment efficacy. Notably, sex differences have garnered increasing attention (6, 7).

Existing research presents conflicting evidence regarding sex-based disparities in EVT outcomes. While multiple studies suggest women experience poorer functional independence and higher post-procedural mortality rates, which may be attributable to several sex-specific factors (8–10). Epidemiological studies highlight that women tend to present with more severe strokes at older ages (11, 12) and have higher pre-stroke disability rates (6). Moreover, sexually biological differences appear to play a significant role, including sexually dimorphic cerebrovascular anatomy and hormone-mediated vascular responses that may influence treatment efficacy (13, 14). Furthermore, sociodemographic disparities in access to acute stroke care and post-stroke rehabilitation may explain significant variations in patient outcomes (15, 16). However, this view is not universal, as other studies have reported comparable outcomes between the sexes following EVT (7, 17, 18). Importantly, overreliance on small-sample studies, which are prone to selection bias and inadequate confounder adjustment, may lead to overestimation or misinterpretation of sex differences in EVT outcomes.

Using the data from the DEVT, RESCUE BT, and MARVEL trials (19–21). This study sought to investigate sex-based differences in outcomes by comparing clinical outcomes between men and women undergoing EVT.

## Methods

### Study population and design

The AIS patients who underwent EVT treatment for anterior circulation LVO were selected from three randomized controlled trials, including the Direct Endovascular Thrombectomy versus Combined IVT and Endovascular Thrombectomy for Patients with Acute Large Vessel Occlusion in the Anterior Circulation (DEVT trial) (19), the Endovascular Treatment With versus Without Tirofiban for Patients with Large Vessel Occlusion Stroke (RESCUE BT trial) (20), and the Methylprednisolone as Adjunctive to Endovascular Treatment for Acute Large Vessel Occlusion (MARVEL trial) (21) (Supplementary Figure 1). The three trials followed the ethical principles of the Helsinki Declaration and were approved by the ethics committees of the Xinqiao Hospital, Army Medical University, and all participating centers. Written informed consent was taken from all patients or their legal representatives before they were enrolled in these trials. The inclusion criteria, data collection, methods, and radiological assessment of the three trials were reported previously.

### Data collection

The following details of the patients were extracted from the combined database: sex, age, and stroke risk factors, including coronary heart disease, atrial fibrillation, valvular heart disease, hypertension, diabetes mellitus, hyperlipidemia, transient ischemic attack, cerebral infarction, intracranial hemorrhage, and smoking history. Furthermore, the cerebral artery occlusion site, baseline glucose, and stroke etiology by the Trial of ORG 10172 in Acute Stroke Treatment (TOAST) classification (22), baseline National Institutes of Health Stroke Scale (NIHSS) score (23), systolic blood pressure upon admission, the American Society of Interventional and Therapeutic Neuroradiology/Society of Interventional Radiology (ASITN/SIR) collateral vessel grading system (24), the Alberta Stroke Program Early Computed Tomography Score (ASPECTS) (25), time from stroke onset to puncture (OTP), and onset to recanalization (OTR) were all included. Two researchers independently collected the data, and any discrepancies were resolved through consensus. If agreement could not be reached, a third researcher was consulted. The quality of reperfusion was evaluated by the expanded Thrombolysis in Cerebral Infarction (eTICI) grade on the final angiogram (26).

### Outcome measures

The primary outcome was defined as the 90-day ordinal modified Rankin Scale score (mRS) distribution. The mRS score ranges from 0 (indicating no residual symptoms) to 6 (indicating death) (27). Secondary outcomes included the favorite outcome (mRS 0 to 3), functional independence (mRS 0 to 2), and excellent outcome (mRS 0 to 1). The safety outcomes were symptomatic intracranial hemorrhage (sICH), assessed according to the Heidelberg bleeding classification (28), mortality within 90 days, and any intracranial hemorrhage.

### Statistical analysis

The variable’s statistics were computed based on the data type. The continuous variables were presented as a median and interquartile range (IQR). Categorical variables were reported as numbers and percentages. The differences between the men and women groups were compared using the Mann–Whitney U-test for continuous and ordinal variables and the Pearson’s χ2 test or Fisher’s exact test for categorical variables.

Furthermore, for the analysis of binary outcomes, binary logistic regression was used, such as mRS 0 to 3, mRS 0 to 2, mRS 0 to 1, sICH, mortality within 90 days, and any intracranial hemorrhage. For ordinal outcomes (e.g., modified Rankin Scale scores), ordinal logistic regression was performed, and results are reported as common odds ratios (common ORs). For binary outcomes, binary logistic regression was used, and results are reported as odds ratios (ORs). Based on the prior literature and the unique characteristics of the present dataset, the following covariates were adjusted for in the analysis: age, atrial fibrillation, hypertension, diabetes, baseline ASPECTS, baseline NIHSS, occlusion site, stroke etiology, anesthesia, and intravenous thrombolysis.

To reduce bias between the women’s and men’s groups, we performed a propensity score matching (PSM) analysis. Using the nearest-neighbor matching algorithm, the covariates were balanced 1:1 with a caliper width of 0.1. PSM used the same adjustment factors as logistic regression. In the matched population, sex-based outcome differences were quantified using binary and ordinal logistic regression models, with results reported as ORs and common ORs.

Subgroup analyses was performed by testing the interaction effect using the binary logistic regression model to investigate sex modification on the primary outcome in the following subgroups from the post-matched population: age (age <75 years versus age ≥75 years), baseline NIHSS (<15 versus ≥15), baseline ASPECTS (<6 versus ≥6), pretreatment with intravenous thrombolysis (yes versus no), stroke etiology (large artery atherosclerosis versus cardioembolism versus other or unknown etiology), usage of tirofiban (yes versus no), and OTP (>340 min versus ≤340 min).

For statistical assessment, IBM SPSS Statistics 26 (SPSS Inc.) and R version 4.4.2 (R Foundation for Statistical Computing, Vienna, Austria) were used. All tests were two-sided and considered statistically significant at a *p*-value of <0.05. A simple imputation method was used for imputing missing values (Supplementary Method 2).

## Results

### Baseline characteristics

Of the patients, 2,862 were included in the analysis. There were 1,641 (57.3%) men and 1,221 (42.7%) women. Women were older (median 72 [IQR, 64–78] versus 65[IQR, 56–73], *p* < 0.001) and had a lower rate of smoking (1.1% versus 45.3%, *p* < 0.001). However, women had a higher prevalence of cardiac heart disease (23.8% versus 14.1%, *p* < 0.001), atrial fibrillation (52.4% versus 30.2%, *p* < 0.001), and diabetes (21.9% versus 18.5%, *p* < 0.001). Women had higher stroke severity by median (IQR) NIHSS score at presentation (18[16–21] versus 17[14–20], *p* < 0.001), higher baseline glucose (7.10[6.30–8.90] versus 7.00[5.90–8.10], *p* < 0.001), shorter median (IQR) onset to puncture time (316[217–540] versus 366[235–619], *p* < 0.001), and shorter median (IQR) onset to recanalization time (400[290–615] versus 454[315–715], *p* < 0.001). The prevalence of stroke subtype according to TOAST criteria was significantly different between groups (*p* < 0.001). LAA was higher in men (49.5% versus 26.5%), while cardioembolism was higher in women (62.0% versus 36.3%). There were significant differences in the first choice of EVT (*p* < 0.001). The use of stent thrombectomy was lower in women (22.4% versus 24.0%). Aspiration was higher in women (46.8% versus 41.0%). Solitaire stent with intracranial support catheter for mechanical thrombectomy (SWIM) was higher in men (20.6% versus 22.9%). Other detailed patient characteristics are presented in Table 1. A total of 1,029 men were successfully matched with 1,029 women presenting with large vessel occlusion. After 1:1 PSM, baseline characteristics were well-balanced between the two groups, except for a history of coronary heart disease and smoking status (Table 1; Supplementary Method 1).

**Table 1:** Baseline and procedural characteristics between women and men.

Characteristic	Prematched population (*n* = 2,862)			Postmatched population (*n* = 1,576)		
	Women (*n* = 1,221)	Men (*n* = 1,641)	*p*-value	Women (*n* = 1,029)	Men (*n* = 1,029)	*p*-value
Age, y, median (IQR)	72 (64–78)	65 (56–73)	<0.001	71 (62–77)	70 (61–76)	0.160
SBP, mmHg, median, (IQR)	144 (127–161)	145 (128–160)	0.453	143 (126–161)	146 (130–161)	0.845
Glucose, mmol/L, median, (IQR)	7.1 (6.3–8.9)	7.0 (5.9–8.1)	<0.001	7.1 (6.3–8.8)	7.0 (6.0–8.2)	0.138
Risk factors, *n* (%)						
Cardiac heart disease	291 (23.8)	232 (14.1)	<0.001	228 (22.2)	181 (17.6)	0.011
Atrial fibrillation	640 (52.4)	496 (30.2)	<0.001	491 (47.7)	469 (45.6)	0.353
Hypertension	730 (59.8)	964 (58.7)	0.601	612 (59.5)	604 (58.7)	0.754
Hyperlipidemia	285 (23.3)	399 (24.3)	0.576	249 (24.2)	222 (21.6)	0.172
Diabetes	267 (21.9)	304 (18.5)	0.03	225 (21.9)	204 (19.8)	0.278
Cerebral infarction	199 (16.3)	248 (15.1)	0.417	168 (16.3)	175 (17.0)	0.723
Smoking	13 (1.1)	743 (45.3)	<0.001	10 (1.0)	433 (42.1)	<0.001
Baseline ASPECTS, median, (IQR)	7 (5–8)	7 (5–8)	0.496	7 (5–8)	7 (5–8)	0.578
Baseline NIHSS, median, (IQR)	18 (16–21)	17 (14–20)	<0.001	18 (15–21)	17 (15–21)	0.845
ASITN/SIR grade, *n* (%)			0.059			0.093
0–1	451 (36.9)	548 (33.4)		360 (35.0)	398 (35.4)	
2	463 (37.9)	623 (38.0)		396 (38.5)	396 (38.5)	
3–4	307 (25.1)	470 (28.6)		273 (26.5)	235 (22.8)	
Occlusion site, *n* (%)			0.042			0.882
Intracranial segment of ICA	356 (29.2)	456 (27.8)		306 (29.7)	297 (28.9)	
MCA M1 segment	687 (56.3)	990 (60.3)		583 (56.7)	594 (57.7)	
MCA M2 segment	178 (14.6)	195 (11.9)		140 (13.6)	138 (13.4)	
Stroke etiology, *n* (%)			<0.001			0.915
Large artery atherosclerosis	323 (26.5)	813 (49.5)		322 (31.3)	332 (32.3)	
Cardioembolism	757 (62.0)	596 (36.3)		580 (56.4)	564 (54.8)	
Other	29 (2.4)	78 (4.8)		29 (2.8)	30 (2.9)	
Unknow	112 (9.2)	154 (9.4)		98 (9.5)	103 (10.0)	
Procedural time variables, min, median, (IQR)						
Onset to puncture	316 (217–540)	366 (235–619)	<0.001	325.0 (222.0–555.0)	320.0 (215.0–538.0)	0.508
Onset to recanalization	400 (290–615)	454 (315–715)	<0.001	408.0 (296.0–630.0)	401.0 (291.0–630.0)	0.732
First choice of EVT, *n* (%)			0.02			0.140
Stent thrombectomy	273 (22.4)	394 (24.0)		228 (22.2)	249 (24.2)	
Aspiration	571 (46.8)	673 (41.0)		479 (46.6)	444 (43.1)	
SWIM	252 (20.6)	376 (22.9)		210 (20.4)	240 (23.3)	
Others	125 (10.2)	198 (12.1)		112 (10.9)	96 (9.3)	
Tirofiban, *n* (%)	427 (35.0)	720 (43.9)	<0.001	382 (37.1)	385 (37.4)	0.927
General anesthesia, *n* (%)	872 (71.4)	1,143 (69.7)	0.326	733 (71.2)	726 (70.6)	0.771
IVT, *n* (%)	337 (27.6)	435 (26.5)	0.543	274 (26.6)	290 (28.2)	0.459
Successful reperfusion^a^, *n* (%)	1,093 (89.5)	1,485 (90.5)	0.423	921 (89.5)	936 (91.0)	0.299

ICH, intracerebral hemorrhage; mRS, modified Rankin Score; IQR, interquartile range; SBP, baseline systolic pressure; ASPECT, Alberta Stroke Program Early CT Score; ASITN/SIR, American Society of Interventional and Therapeutic Neuroradiology/ Society of Interventional Radiology; NIHSS, National Institutes of Health Stroke Scale score; ICA, internal carotid artery; M1, M1 segment of the middle cerebral artery; M2, M2 segment of the middle cerebral artery; IVT, intravenous thrombolysis; EVT, endovascular treatment; SWIM, Solitaire stent with intracranial support catheter for mechanical thrombectomy.

a Successful reperfusion represents eTICI 2b50 and above.

### Clinical outcomes

**Figure 1:**
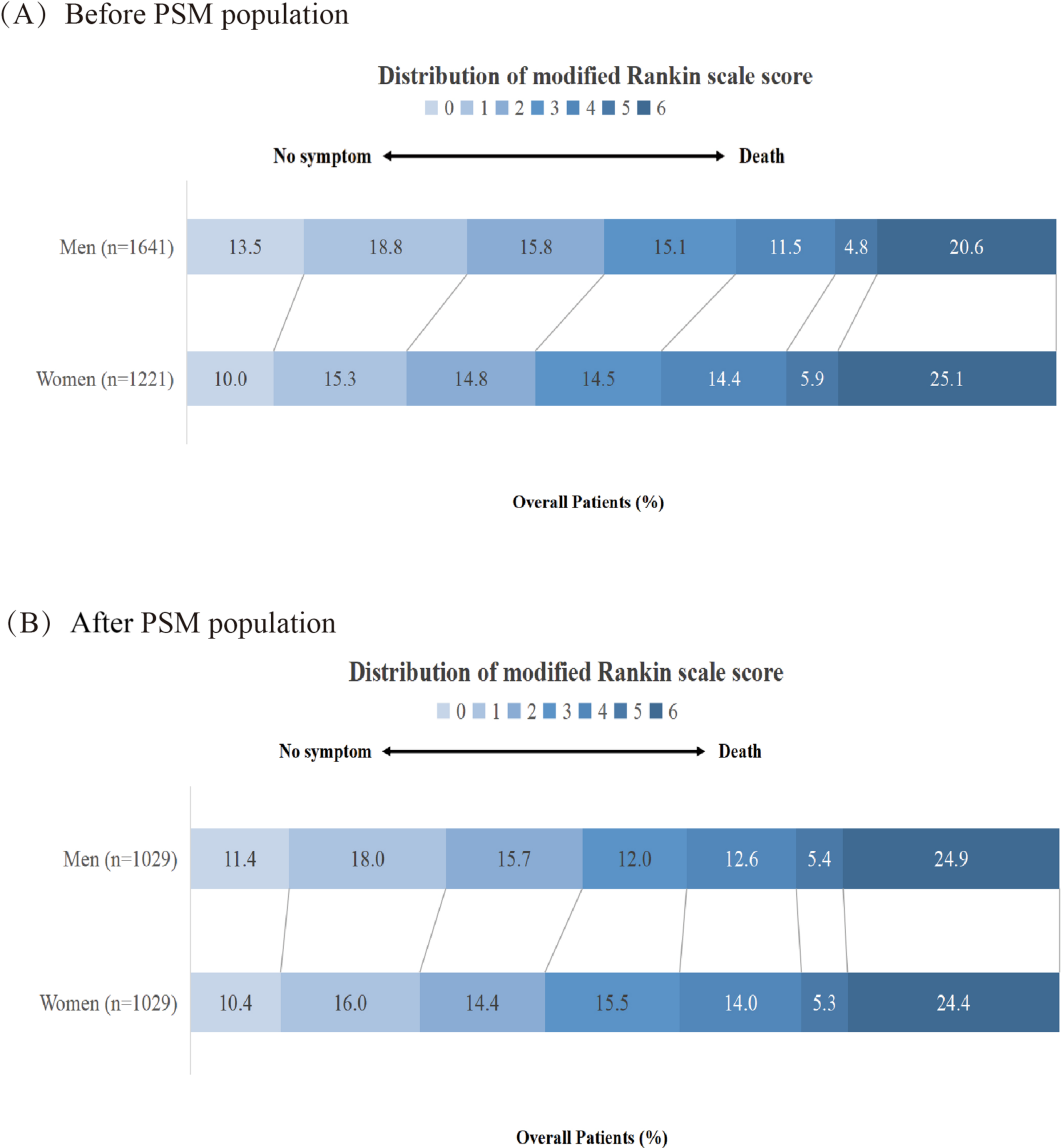
Shift in 90-day modified Rankin scale (mRS) score stratified by women and men in the pre- and postmatched population. PSM, propensity score matching. **(A)** represents the before PSM population. **(B)** represents the after PSM population.

Table 2 provides primary, secondary, and safety outcome comparisons between men and women. Before adjusting for the confounders, we found that men had a shift to better outcomes (3[1–6] versus 3[1–5], common OR, 1.37[1.21–1.57], *p* < 0.001; Figure 1A), higher odds of favorable outcome (54.6% versus 63.1%, OR 1.42[1.22–1.65], *p* < 0.001), higher odds of functional independence (40.1% versus 48.1%, OR, 1.38[1.19–1.60], *p* < 0.001), higher odds of excellent outcome (25.3% versus 32.2%, OR, 1.40[1.19–1.66], *p* < 0.001), and lower odds of mortality (25.1% versus 20.6%, OR, 0.78[0.65–0.93], *p* = 0.005) than women.

**Table 2:** Outcomes comparison between women and men.

Outcomes	Prematched population (*n* = 2,862)						Postmatched population (*n* = 2058)			
	Women (*n* = 1,221)	Men (*n* = 1,641)	Unadjusted analysis		Adjusted analysis		Women (*n* = 1,029)	Men (*n* = 1,029)	PS-score adjusted analyses	
			Effected (95%CI)	*p*-value	Effected (95%CI)	*p*-value			Effected (95%CI)	*p*-value
Primary outcome										
90-day mRS^b^, median, IQR	3 (1–6)	3 (1–5)	1.37 (1.21–1.57)	*P* < 0.001	1.02 (0.89–1.18)	0.741	3 (1–5)	3 (1–5)	1.03 (0.88–1.20)	0.712
Secondary outcome										
mRS0-3^a^	667 (54.6)	1,036 (63.1)	1.42 (1.22–1.65)	*P* < 0.001	1.00 (0.84–1.19)	0.988	452 (57.4)	465 (59.1)	0.98 (0.81–1.19)	0.874
mRS0-2^a^	490 (40.1)	789 (48.1)	1.38 (1.19–1.60)	*P* < 0.001	1.12 (0.94–1.32)	0.211	322 (40.9)	356 (45.2)	1.18 (0.98–1.43)	0.079
mRS0-1^a^	309 (25.3)	529 (32.2)	1.40 (1.19–1.66)	*P* < 0.001	1.11 (0.92–1.34)	0.273	205 (26.0)	231 (29.4)	1.15 (0.93–1.41)	0.195
Safety outcome										
sICH^a^	109 (8.9)	151 (9.2)	1.03 (0.80–1.34)	0.800	1.23 (0.94–1.63)	0.134	73 (9.3)	82 (10.4)	1.24 (0.92–1.67)	0.163
Mortality within 90 days^a^	306 (25.1)	338 (20.6)	0.78 (0.65–0.93)	0.005	1.04 (0.86–1.27)	0.670	189 (24.0)	186 (23.6)	1.07 (0.86–1.32)	0.542
Any ICH^a^	426 (34.9)	553 (33.7)	0.95 (0.81–1.11)	0.507	1.07 (0.91–1.27)	0.414	276 (35.1)	293 (37.2)	1.05 (0.88–1.27)	0.576

mRS, modified Rankin Score; CIs, confidence intervals; IQR, interquartile range; ICH, intracerebral hemorrhage; sICH, symptomatic intracerebral hemorrhage.

a The odds ratios were estimated from a binary logistic regression model.

b The odds ratios were estimated from an ordinal regression model.

After adjusting for confounders, all outcomes were similar between men and women. After 1:1 PSM, there was no difference in primary, secondary, and safety outcomes. In the primary outcomes, the shift to mRS between women and men showed no difference (3[1–5] versus 3[1–5], common OR, 1.03[0.88–1.20]; *p* = 0.712; Figure 1B). In the secondary outcomes, mRS 0 to 3 was observed in 579 of 1,029 (56.3%) women and 587 of 1,029 (57.0%) men (OR, 0.98[0.81–1.19], *p* = 0.874), mRS 0 to 2 was observed in 420 (40.8%) of women and 464 (45.1%) of men (OR,1.24[0.99–1.55], *p* = 0.079), and mRS 0 to 1 was observed in 272 (26.4%) of women and 302 (29.3%) of men (OR,1.15[0.93–1.51], *p* = 0.195). In the safety outcomes, sICH was observed in 90 (8.7%) of women and 108 (10.5%) of men (OR,1.24[0.92–1.67], *p* = 0.162), mortality was observed in 251 (24.4%) of women and 256 (24.9%) of men (OR,1.07[0.86–1.32], *p* = 0.542), and any intracranial hemorrhage was observed in 360 (35.0%) of women and 371 (36.1%) of men (OR,1.05[0.88–1.27], *p* = 0.576).

### Subgroup analysis

**Figure 2:**
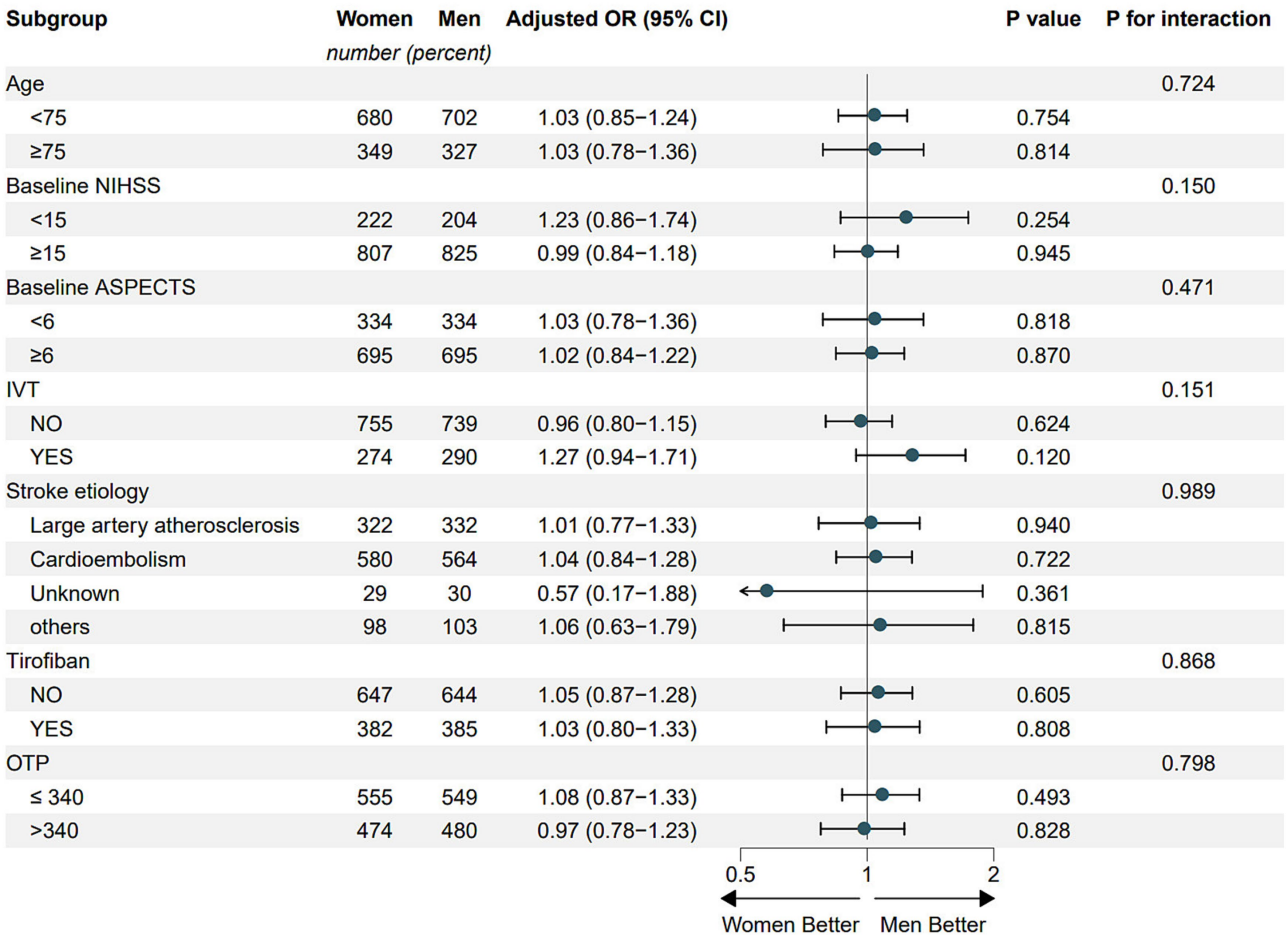
Subgroup analysis. NIHSS, National Institutes of Health Stroke Scale; ASPECTS, Alberta Stroke Program Early Computed Tomography Score; IVT, intravenous thrombolysis; OTP, onset to puncture.

The shift to mRS at 90 days remained consistent across the pre-defined subgroups, including age (age <75 years versus age ≥75 years), baseline ASPECTS (<6 versus ≥6), pretreatment with intravenous thrombolysis (yes versus no), stroke etiology (large artery atherosclerosis versus cardio embolism versus other or unknown etiology), usage of tirofiban (yes versus no), and OTP (>340 min versus ≤340 min) (Figure 2). There was no interaction between sex and pre-defined subgroup in terms of the treatment effect modification in EVT outcomes.

### Marginal effect and outcomes

**Figure 3:**
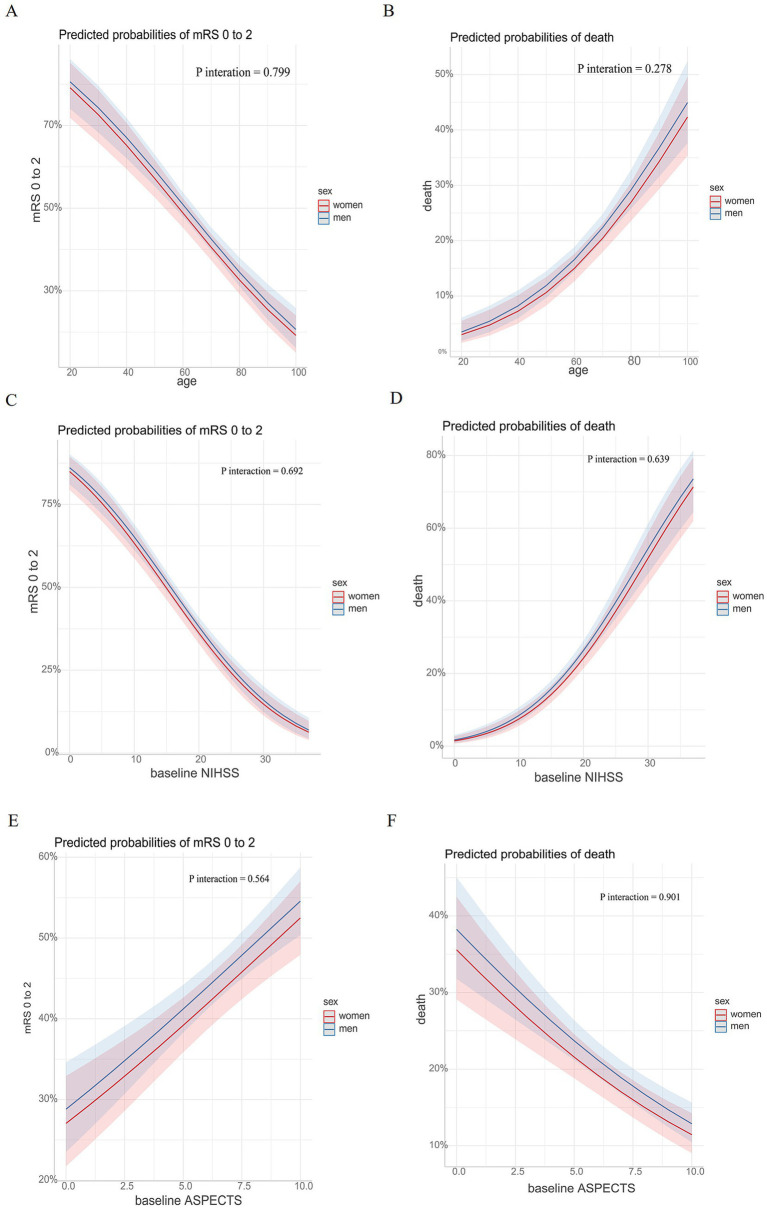
Marginal effects and clinical outcomes. Association of age, baseline NIHSS, and baseline ASPECTS with the probability of clinical outcomes. The probabilities of experiencing an mRS score of 0 to 2 based on age, baseline NIHSS, and baseline ASPECTS among patients with anterior circulation large vessel occlusion are presented in **(A,C,E)**. The probabilities of experiencing death based on age, baseline NIHSS, and baseline ASPECTS among patients with anterior circulation large vessel occlusion are presented in **(B,D,F)**. NIHSS, National Institutes of Health Stroke Scale; ASPECTS, Alberta Stroke Program Early Computed Tomography Score; mRS, modified Rankin Scale.

We analyzed the association of age, baseline NIHSS score, and baseline ASPECTS with clinical outcomes (defined as an mRS score of 0 to 2 and mortality) among patients stratified by sex. The results showed that for both men and women, the predicted probability of achieving an mRS score of 0–2 gradually decreased with increasing age and baseline NIHSS score but increased with higher baseline ASPECTS. In contrast, the predicted probability of mortality showed the opposite trend. No interaction effects were observed among the different subgroups (Figure 3).

## Discussion

Based on the DEVT, RESCUE BT, and MARVEL databases, a large sample size of 2,862 patients was selected, and baseline and outcome data were analyzed. Our findings demonstrated that there were no significant differences in clinical or safety outcomes between men and women following EVT for anterior circulation LVO. Additionally, no interaction was observed between sex and predefined subgroups regarding the treatment effect of modification in EVT outcomes.

This study revealed significant gender disparities in baseline demographic characteristics and clinical presentations. Notably, fewer women underwent EVT compared to men. Previous studies have suggested that the reduced utilization of EVT in women compared to men may be attributable to their less favorable baseline characteristics, including a higher prevalence of living alone, advanced age, and greater pre-stroke disability (29). In our study, we found that women patients were not only significantly older than men patients but also exhibited markedly higher prevalence rates of cardiac heart disease, atrial fibrillation, and valvular pathologies. This distinct clinical profile was associated with a higher incidence of cardioembolic events in women, which may potentially explain the observed shorter endovascular treatment procedure times compared to men, consistent with findings from recent studies (7, 30). In contrast, men showed significantly higher rates of smoking and tandem lesions, with large artery atherosclerosis being the predominant etiology. The distinct vascular risk factor profiles observed between groups in the pre-matched cohort may account for the differential pathophysiology of large vessel occlusion.

Other than baseline demographic characteristics and clinical presentations, sex-specific factors may affect the differences in post-EVT outcomes. A questionnaire study revealed that women who arrived at the hospital more than 3 h after symptom onset were significantly more likely to report reluctance to trouble others and adopt a “wait-and-see” approach for potential symptom resolution and were more likely to rest, take medication to treat symptoms, hide symptoms from others, and continue with usual activities (31). Additionally, women are at higher risk for poststroke depression and have reduced mobility compared with men, which may significantly impact their post-intervention rehabilitation outcomes (32). Moreover, based on the cardiovascular protective effects of endogenous estrogen, several studies have investigated the relationship between female reproductive lifespan (defined as menopause age minus menarche age) and ischemic stroke risk (33, 34). Research consistently shows that women with shorter reproductive lifespans face a significantly increased risk of developing ischemic stroke (35, 36).

Consistent with prior evidence, our analysis revealed that there was no interaction between sex and pre-defined subgroup in terms of the treatment effect modification in EVT outcomes (37). While a recent late-window EVT study similarly found no sex-based differences in functional outcomes, they reported age-dependent risks in male patients for mortality (*P*_interaction_ = 0.003) and symptomatic intracranial hemorrhage (*P*_interaction_ = 0.017), an effect not observed in our cohort (mortality: *p* = 0.534; sICH: *p* = 0.685; Supplementary Figures 2, 3). The authors attributed these findings to biological, genetic, and socio-economic factors (38). Another study showed that in patients with an NIHSS score of <15, women tended to have a better outcome than men, whereas there was no gender effect in those with an NIHSS score of ≥15. Regrettably, the research did not pursue mechanistic studies to explain these sex-specific outcome patterns (30).

However, with improved health care coverage, optimized treatment protocols, significantly enhanced public stroke awareness, and strong governmental policy support, women have demonstrated greater improvements in outcomes compared to men over the past decade (39). Moreover, the absence of sex-based disparities in functional outcomes may be attributed to improved stroke awareness among younger women, leading to earlier diagnosis, more timely treatment, enhanced rehabilitation access, and ultimately superior clinical outcomes (34). Additionally, women exhibit distinct cerebrovascular anatomical characteristics, including smaller arterial diameters, higher tortuosity indices, and more robust collateral circulation (40). They may benefit from the use of less traumatic EVT devices that reduce the risk of vessel perforation, which may not be as deleterious as in men, given their better collateral circulation (9).

## Limitation

There are some limitations to our study. First, as this is a pooled analysis of three randomized controlled trials, all participants were selected under strict trial criteria. This may result in differences between our study sample and the broader real-world EVT population in terms of age, comorbidity burden, and workflow times. Second, only data from patients with an anterior circulation LVO who received EVT were evaluated, so we cannot determine whether similar sex differences exist among posterior circulation LVO stroke patients. Third, our study focused primarily on evaluating outcomes within the 90-day post-treatment window, a time point widely recognized as clinically meaningful for assessing recovery and functional outcomes in ischemic stroke. The existence of sex-based differences in long-term functional outcomes following EVT remains an area of active investigation, with current findings presenting inconsistencies. Therefore, caution is warranted when generalizing the conclusions of this study to all anterior circulation large-vessel occlusion patients in routine clinical practice. Future studies are needed to validate these findings in real-world cohorts.

## Conclusion

This pooled analysis demonstrated that no statistically significant differences were observed between men and women in clinical or safety outcomes, following EVT for anterior circulation LVO. Furthermore, there was no evidence of an interaction between sex and predefined subgroups in terms of treatment effect modification for EVT outcomes.

## Data Availability

The data analyzed in this study is subject to the following licenses/restrictions: the data that support the findings of this study are available from the corresponding author upon reasonable request. Requests to access these datasets should be directed to LZ 17861523212@163.com.
