# Supplementary material for: Sex-based differences in outcomes following endovascular therapy for anterior circulation large vessel occlusion: a pooled analysis of DEVT, RESCUE BT, and MARVEL trials
Source: Front Neurol. 2026 Jan 12;16:1753257. doi: 10.3389/fneur.2025.1753257 (PMC12832441; doi:10.3389/fneur.2025.1753257)
Supplement: Supplementary file 1 [file Table_1.DOCX]

**Supplement materials**

**Supplement Method 1.** Propensity score matching analysis.

**Supplement Method 2** Imputation method.

**Supplement Figure 1.** Follow chart.

**Supplement Figure 2.** Subgroup analysis of sICH stratified by sex after propensity score matching.

**Supplement Figure 3.** Subgroup analysis of mortality within 90 days stratified by sex after propensity score matching.

This supplementary material has been provided by the authors to give readers additional information about their work.

**Supplement Method 1.** Propensity score matching analysis.

We performed a 1:1 propensity score matching based on the nearest–neighbor matching algorithm with a caliper width of 0.1 using R (R Core Team. R: A Language and Environment for Statistical Computing. R Foundation for Statistical Computing. Vienna, Austria, 2021).

**Propensity score matching Variables & Method**

Variables:

- age, atrial fibrillation, hypertension, diabetes, Baseline ASPECTS, Baseline NIHSS, occlusion site, stroke etiology, anesthesia, intravenous thrombolysis.

Matching：

- Method = “nearest”
- distance = “logit”
- ratio = 1
- caliper = .1

| Sample Sizes: | | |
| --- | --- | --- |
|  | Women | Men |
| All | 1221 | 1641 |
| Matched | 1029 | 1029 |
| Unmatched | 192 | 612 |
| Discarded | 0 | 0 |

**Matching Graphs**


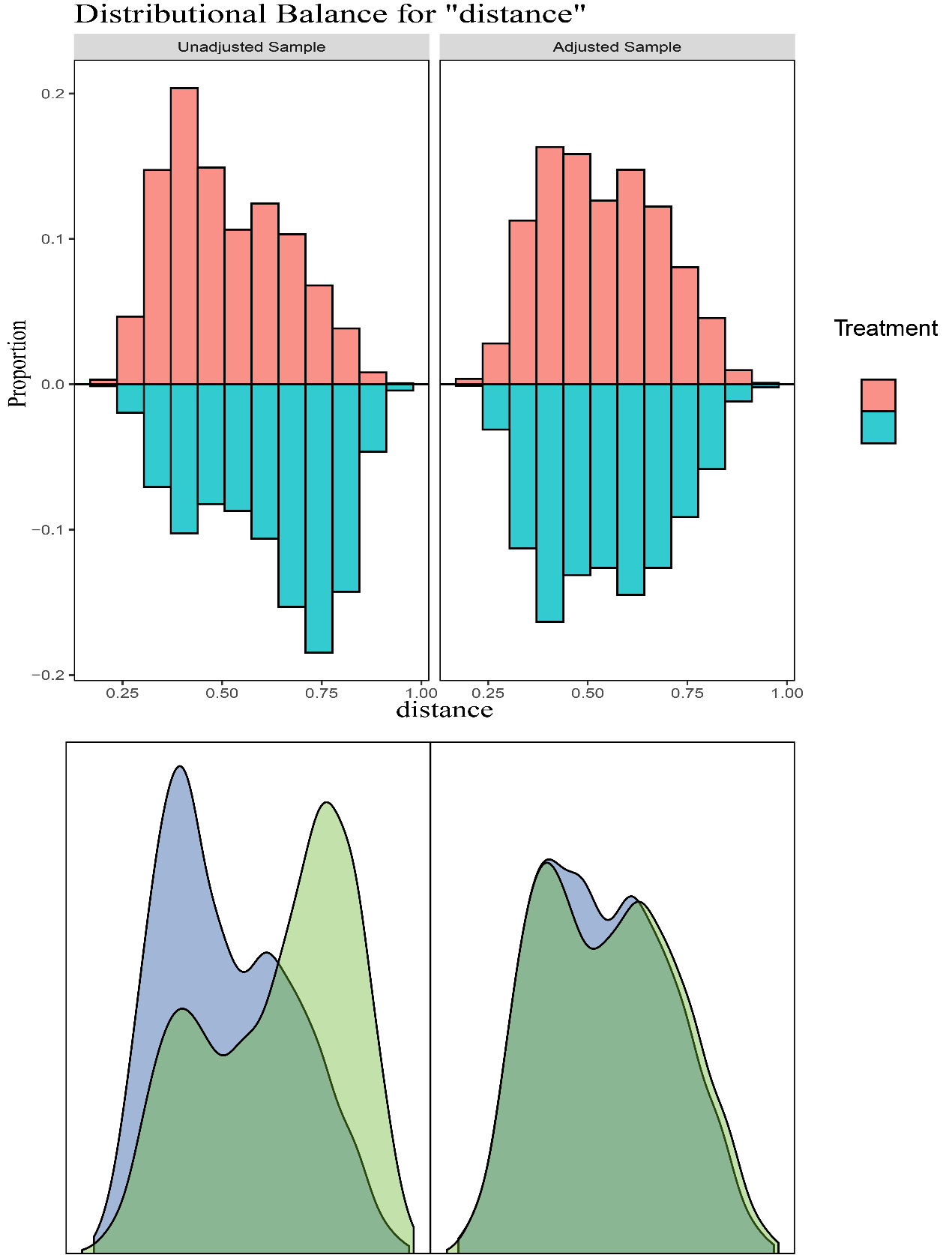


**Supplement Method 2** Imputation method.

All statistical analyses were conducted on the imputed dataset. The missing values were imputed using a simple imputation approach, with the mode for categorical variables and the median for continuous variables. 6 baseline ASPECTS, 19 baseline ASITN/SIR and 278 baseline glucose levels, 1 TOAST, 5 anesthesia, 6 extended thrombolysis in cerebral infarction (eTICI) scale, 7 OTP, 8 OTR time, 33 sICH and any intracranial hemorrhage were imputed. The proportion of missing data remained within an acceptable range (less than 10%).

**Supplement Figure 1.** Follow chart.

**
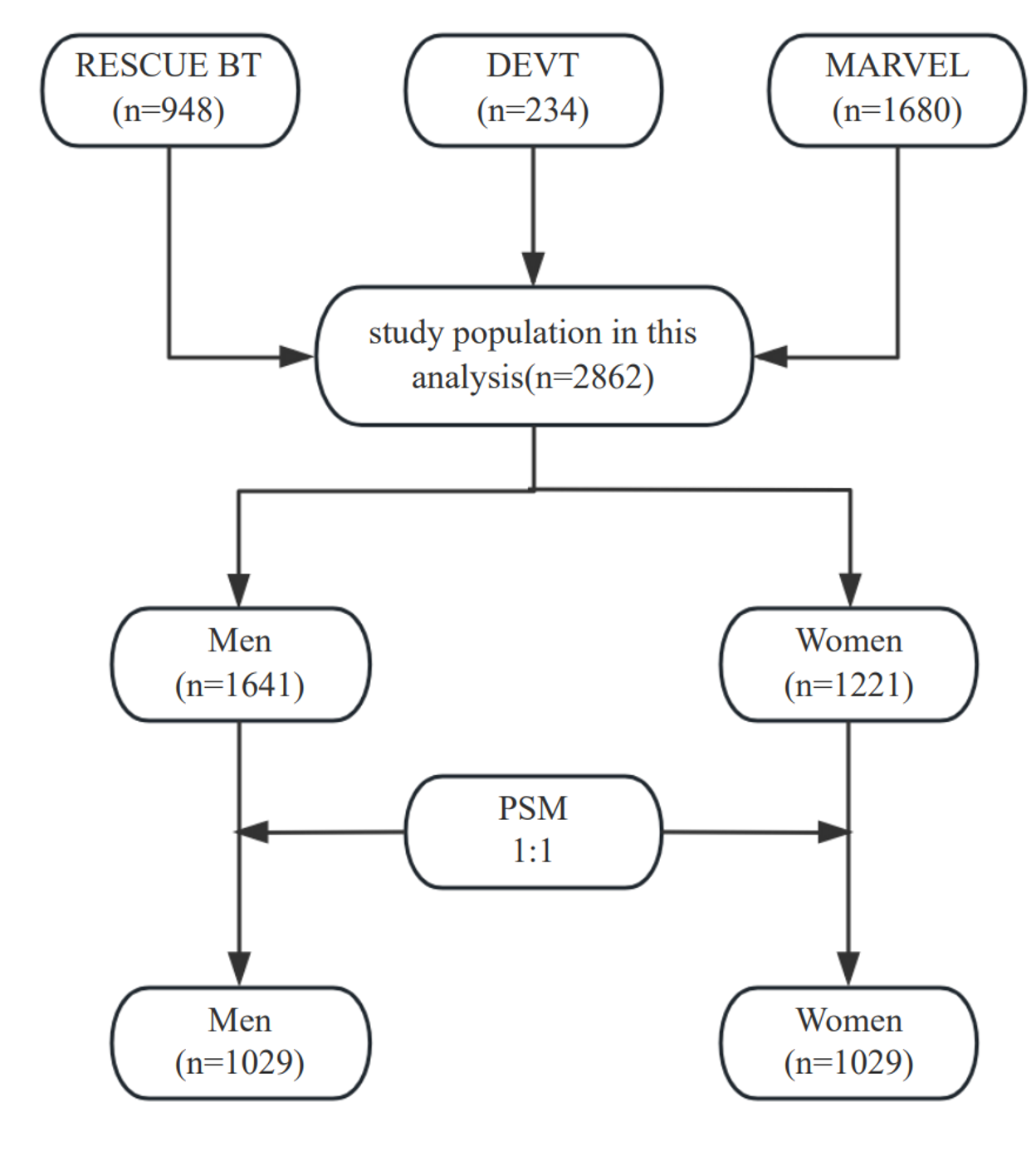
**

Abbreviations: RESCUE BT, The Endovascular Treatment With vs Without Tirofiban for Patients with Large Vessel Occlusion Stroke; DEVT, Direct Endovascular Thrombectomy vs Combined IVT and Endovascular Thrombectomy for Patients with Acute Large Vessel Occlusion in the Anterior Circulation; MARVEL, The Methylprednisolone as Adjunctive to Endovascular Treatment for Acute Large Vessel Occlusion; PSM, propensity score matching.

**Supplement Figure 2.** Subgroup analysis stratified by gender with death as the outcome after propensity score matching.


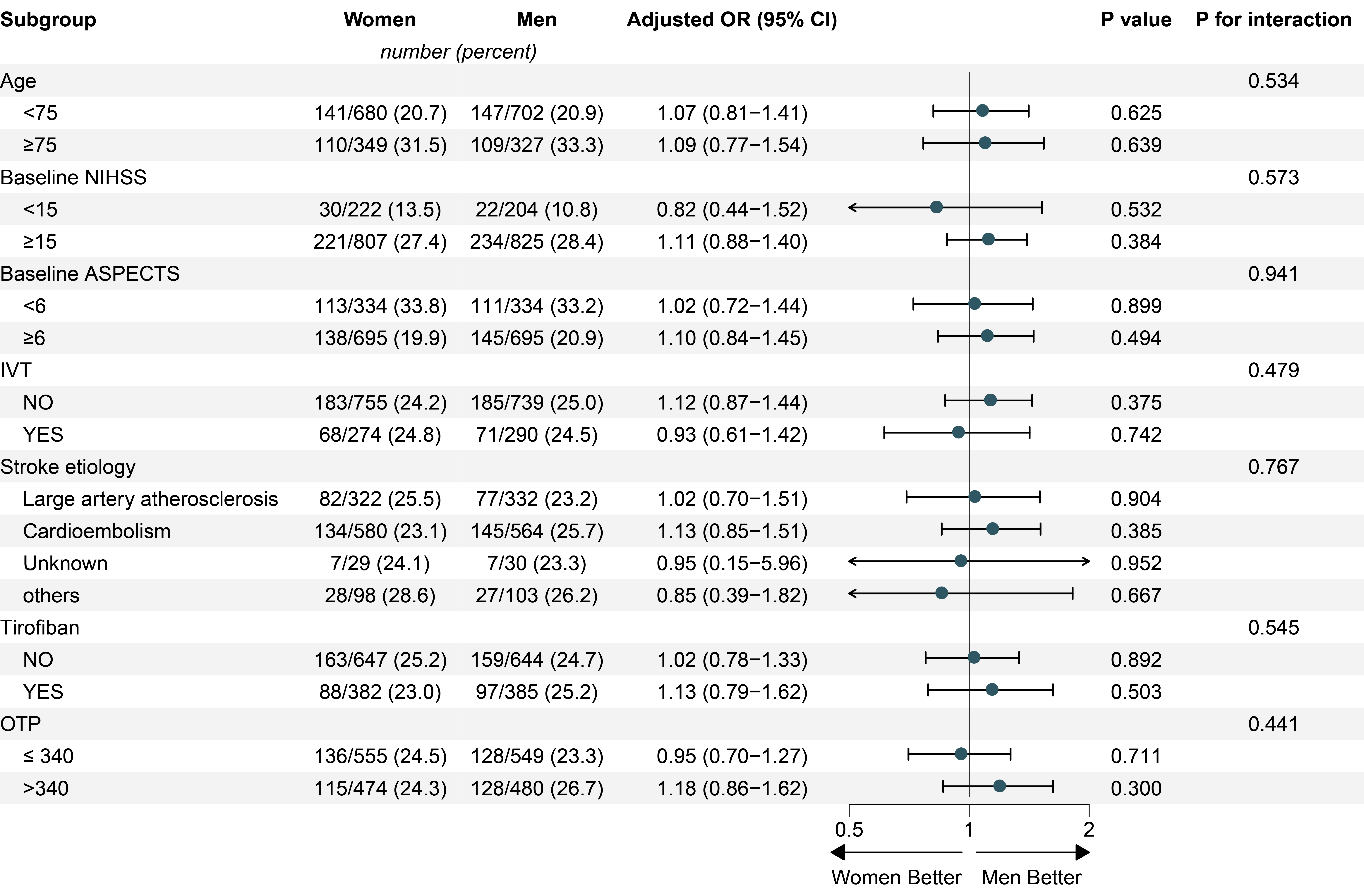


Abbreviations: NIHSS, National Institutes of Health Stroke Scale; ASPECTS, Alberta Stroke Program Early Computed Tomography Score; IVT, intravenous thrombolysis; OTP, onset to puncture.

**Figure S3.** Subgroup analysis stratified by sex with symptomatic intracranial hemorrhage as the outcome after propensity score matching.


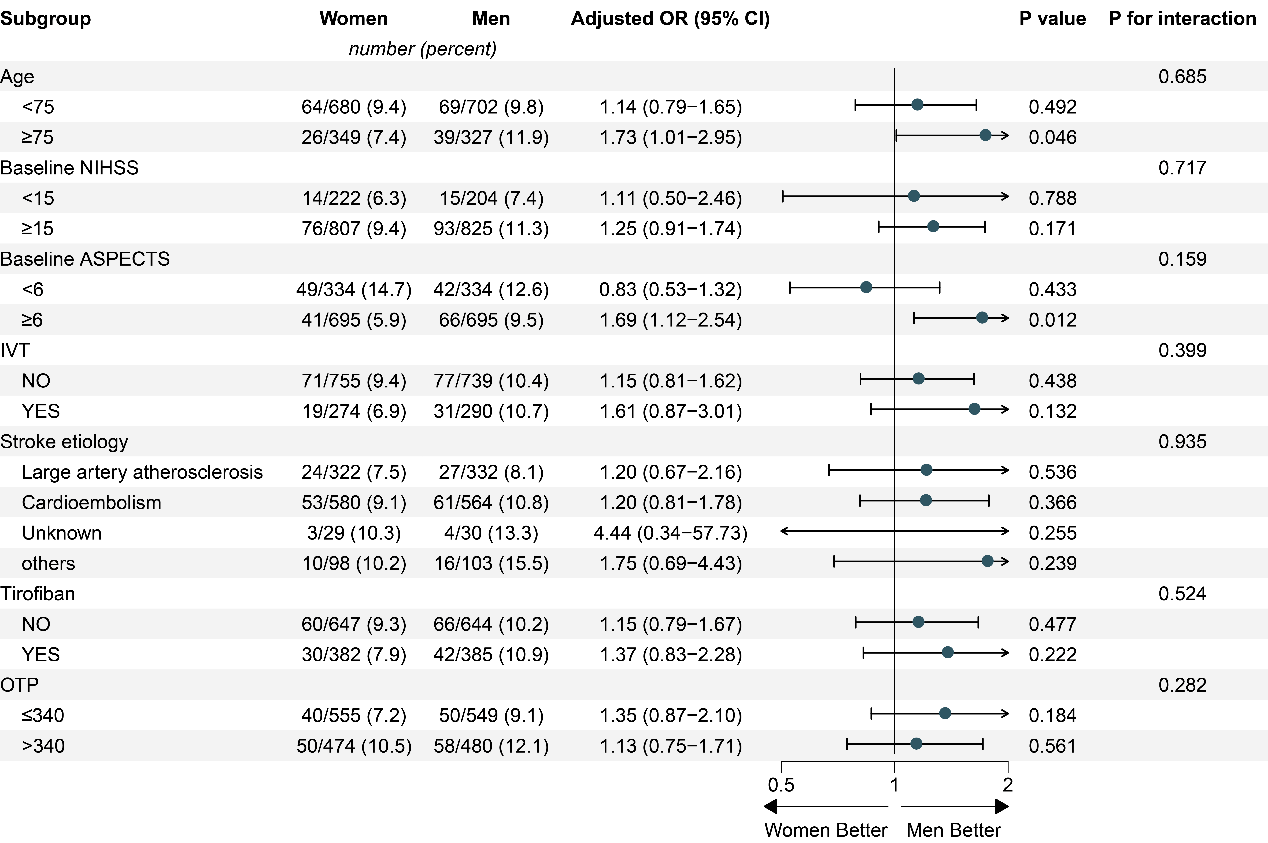


Abbreviations: NIHSS, National Institutes of Health Stroke Scale; ASPECTS, Alberta Stroke Program Early Computed Tomography Score; IVT, intravenous thrombolysis; OTP, onset to puncture.
